# Physical Fighting and Leisure Activities among Norwegian Adolescents—Investigating Co-occurring Changes from 2015 to 2018

**DOI:** 10.1007/s10964-020-01252-8

**Published:** 2020-05-27

**Authors:** Lars Roar Frøyland, Anders Bakken, Tilmann von Soest

**Affiliations:** 1 NOVA–Norwegian Social Research, OsloMet–Oslo Metropolitan University, Oslo, Norway; 2 PROMENTA Research Center, Department of Psychology, University of Oslo, Oslo, Norway

**Keywords:** Time trends, Physical fighting, Violence, Leisure activities

## Abstract

After many years of decline in violent behavior among adolescents in several Western countries, recent official statistics indicate a possible trend change. So far, knowledge on how this change is related to co-occurring changes in leisure time activities is limited. Using two cross-sectional surveys from Oslo, Norway, this study found substantial increases in the prevalence of physical fighting from 2015 (*N* = 23,381; 51.6% girls) to 2018 (*N* = 25,287; 50.8% girls) in junior and senior high school. The rise in fighting was related to co-occurring changes in several leisure activities, including increasing time spent unsupervised by adults, rising digital media use, and rising cannabis use. The study emphasizes the importance of considering leisure time activities when addressing adolescent misbehavior.

## Introduction

Research has found declining levels of violent behavior among adolescents in the last two decades (Arnett 2018). In the last few years, however, a possible trend change has been unveiled. Crime statistics show an increase in violent crime among young people in Norway (The City of Oslo and Oslo Police District 2019) and a rise of crime in general among young men in Sweden (The Swedish National Council for Crime Prevention 2020). In Denmark, the level of registered violent crime in the population is at its highest level since 1995 (Statistics Denmark 2020). Increasing our knowledge of factors related to changes in the prevalence of violence on the societal level is important in view of developing prevention strategies. Co-occurring changes in leisure time activities have been hypothesized to be important for understanding changes in the societal level of adolescent violence (Arnett 2018). However, a lack of suitable data has so far hampered statistical investigations of the observed changes (for exceptions, see Frøyland and von Soest 2018; Salas-Wright et al. 2017). The present study utilized the population-based high school surveys *Young in Oslo 2015* and *Young in Oslo 2018* to investigate changes in physical fighting on the societal level among Norwegian adolescents over a three-year span. Co-occurring changes in a variety of adolescent leisure time activities were investigated as potential contribution factors for the shifting violence rates, among them leisure time both supervised and unsupervised by adults, the use of digital media, and substance use.

### Time Trends in Violent Behavior

In many Western countries, research has identified declining levels of violent behavior among adolescents in past decades. For example, the cross-national survey Health Behaviour in School-Aged Children (HBSC) found a marked decline in physical fighting between 2002 and 2010 among 11- to 15-year olds in 19 of 30 participating countries in Europe and North America, whereas stable trends were found in eight countries, and only three showed an increase (Pickett et al. 2013). The results have been corroborated by studies conducted in the period from 1991 to 2017 in Norway (Frøyland and von Soest 2018), Sweden (Svensson and Ring 2007), and the United States (Centers for Disease Control and Prevention 2018). Alongside the observed decline in violent behavior, other forms of problem behavior among adolescents show similar trends (Arnett 2018).

However, a possible trend change has occurred since 2014–2015, and the police in several countries have issued warnings about increasing levels of violent behavior among both adolescents and in the general population. After a steady decline from 2007 to 2013 in police registered violent crime among adolescents under the age of 18 in Oslo, the capital of Norway, the number of violent crimes increased from 259 to 499 from 2013 to 2018, an increase of 93% in five years (The City of Oslo and Oslo Police District 2019). Danish crime statistics also show a marked increase in violent crime in the general population from 2015 to 2019 (Statistics Denmark 2020), while Swedish crime statistics show a 20% increase in the number of registered suspects of crime among boys in the age group 18–20 years from 2015 to 2019 (The Swedish National Council for Crime Prevention 2020). The police in England and Wales reported a 7% increase in recorded offences involving a knife or sharp instrument from 2018 to 2019, a finding that was co-occurring with a 2% increase in hospitalizations due to injuries related to the use of sharp objects. The prevalence of less serious forms of violent crime was rather stable (Office for National Statistics 2020).

So far, self-report studies on the possible trend changes in violent behavior are scarce. However, the U.S. Youth Risk Behavior Survey (YRBS), which has revealed a steady decline in physical fighting among high school students for over 10 years, showed a small (but statistically non-significant) increase in such behavior from 2015 to 2017 (Centers for Disease Control and Prevention 2018). In total, these figures signal a possible shift in the unitary trend of a decreasing level of violent behavior among adolescents in past decades, although future research has to provide more information about whether reports of increasing prevalence of violent behavior are stable and long-lasting.

### Leisure Activities and Violent Behavior

One of the most prominent theoretical frameworks for understanding crime rate trends is the Routine Activity Approach (Hollis et al. 2013), which hypothesize that one key element for crime to be likely to occur in a given time and space is the absence of capable guardians, such as the absence of adults in the vicinity when adolescents behave aggressively, combined with the presence of a likely offender and a suitable target. This study addresses the key role of adult supervision in theoretical models of understanding crime rate trends by examining how changes in leisure time patterns are related to adolescent physical fighting, because leisure time activities arguably are the most important domain in adolescents’ life where adult supervision is changing.

In line with theoretical approaches, researchers have shown that time spent unsupervised by adults (Hoeben et al. 2016) and low parental knowledge of adolescents’ activities (Flanagan et al. 2019) are important individual level risk factors for adolescent crime. Unstructured leisure time spent with peers without an authority figure present has also been related to individual offending, through placing adolescents in a situation with a lack of adult social control and no presence of capable guardians (Haynie and Osgood 2005). Adult supervision during adolescent leisure time has seen marked changes in recent years. Whereas spending time out with friends without adult supervision was an important part of being young 20 years ago, U.S. adolescents now spend significantly more of their leisure time at home, an arena where adults most often are present (Twenge 2017). Organized leisure activities are another arena for spending leisure time under adult supervision, but research on trends in adolescent participation in organized activities is scarce. However, a recent study found no change in adolescent participation in school-based extracurricular activities the last three decades (Meier et al. 2018), which is in line with findings from older studies on general participation in organized leisure activities (Mahoney et al. 2006).

An issue related to the time adolescents spend under adult supervision is their use of digital media. One line of reasoning is that rising media use among adolescents have led to a decline in unstructured socializing, which again has contributed to a decline in problem behavior (Arnett 2018). Whereas adolescents 50 years ago rarely spent time in this manner, screen time is the most prominent leisure time activity among young people today (Twenge et al. 2018). By staying at home in front of a screen, today’s adolescents spend more time in the vicinity of adults and are exposed to fewer situations that facilitate norm-breaking behavior. However, researchers have suggested that the increased portability of digital media platforms will over time erode the gains achieved regarding trends in adolescent problem behavior (Green 2016).

New patterns of digital media use among adolescents may also increase the risk of problematic behavior. Research has shown that some adolescents use social media to communicate threats, taunts, and intimidations, which in turn is related to real-life violent and criminal behavior (Cannon et al. 2015). The combination of such patterns of use and the fact that social media are increasingly used on smartphones outside the home (Pew Research Center 2018) may render an association between increases in social media use and rising levels of violence possible. No studies have so far investigated the association between trends in social media use and trends in violence, but a recent review identified a positive cross-sectional association between social media use and adolescent aggression (Vannucci et al. 2020).

Finally, co-occurring changes in substance use may be relevant for understanding changes in the rate of adolescent violence on a societal level. Although perhaps not a leisure activity in itself, substance use is a recreational activity among adolescents that can be directly related to the individual propensity of aggressive and violent behavior (Tomlinson et al. 2016) and normally happens without adult presence. Especially important are the intoxicating effects of alcohol, which may result in impaired judgment in situations where violent behavior is a possible action (Tomlinson et al. 2016). Cannabis use is also associated with violent behavior (Liu and Petras 2017), but the association is typically explained as an indirect association caused by an antisocial lifestyle in general or by other confounding variables (Barthelemy et al. 2016). Alcohol use has declined significantly among adolescents in the last two decades (Pape et al. 2018), and previous research has identified a significant association between co-occurring declines in aggressive behavior and alcohol intoxication among Norwegian adolescents (Frøyland and von Soest 2018). A study among US adolescents also included trends in five measures on substance use as control variables for understanding a decline in physical fighting, but the analyses did not single out the effects of substance use from effects of other control variables included in the study (Salas-Wright et al. 2017). Hence, investigation of whether an increase in fighting at the societal level also co-occurs with shifting trends in substance use is warranted.

### Key Background Variables

When analyzing associations between changes in physical fighting and co-occurring changes in leisure time activities, it is of importance to account for simultaneous changes in key background variables that may be underlying drivers of the observed associations. In particular, changes in the share of adolescents with migration background and differences across time in socioeconomic background, academic achievements, and age of participants may account for observed changes in both physical fighting (Salas-Wright et al. 2017) and patterns of leisure time activities (Bartko and Eccles 2003). These key background variables were, therefore, included as control variables in all analyses.

## Current Study

This study investigates changes in the rate of physical fighting between 2015 and 2018 among adolescents in Oslo, Norway, and how potential shifts are related to co-occurring changes in leisure time activities. Three aspects of adolescent leisure time were considered: adult supervision, digital media use, and substance use. Based on recent data from official crime statistics, an increase in physical fighting among adolescents in Oslo from 2015 to 2018 was anticipated. The increase was expected to be related to a range of potential changes in adolescents’ leisure time activities without adult supervision, among them an increase in time spent out with friends, a decrease in time spent at home, less parental knowledge of adolescent activities, and a decline in participation in organized leisure activities. Further, co-occurring changes in digital media use were hypothesized to be related to the anticipated rise in physical fighting. Even though increasing time in front of a screen in older studies is related to trends towards more adult supervision and less violence, the present study might not necessarily show such a relationship because associations of screen use with adult supervision and being at home may have changed in recent years due to the extensive use of smartphones. Finally, increases in alcohol intoxication and cannabis use, if observed, were hypothesized to be related to the expected increase in adolescent physical fighting. Changes in physical fighting may as well be related to potential changes in key background variables, such as years of schooling, socioeconomic background, migration background, and school grades. These background variables were therefore included as control variables in all analyses.

## Methods

### Procedure and Participants

The present study used data from two school-based cross-sectional surveys of adolescents in Oslo, Norway in 2015 and 2018 (*Young in Oslo 2015* and *Young in Oslo 2018*). All junior and senior high schools in Oslo were asked to participate in the surveys. Except for schools for students with special needs or difficulties with the Norwegian language and a few private senior high schools, all Oslo schools accepted the invitation. Students at the participating junior or senior high schools were invited to complete an electronic questionnaire in class, containing questions about their social lives, health, leisure activities, drug use, and misbehavior. In the 2015 survey, 23,381 students participated, yielding a response rate of 79%. The response rates at junior and senior high schools were 86% and 72%, respectively. In 2018, 25,287 students participated, with an overall response rate of 74%. Response rates were 83% and 65% at junior and senior high schools, respectively. As the surveys sampled almost a complete population of students attending high school in Oslo, only adolescents that did not attend high school or were absent from school at the time of the survey did not receive an invitation to participate. In total, approximately two out of three adolescents in the age group 13–18 residing in Oslo participated in the surveys. Students consented to participation by filling out the survey; the parents of students younger than age 18 were given the option to decline their children’s participation. The Norwegian Center for Research Data approved all ethical aspects of the senior high school survey, and the survey was conducted anonymously for the students at junior high schools.

### Measures

#### Physical fighting

Physical fighting was assessed in 2015 by two items from an instrument measuring the frequency of different conduct problems: “How many times have you done any of the following things over the past year (the past 12 months)?” with the items “have been in a fight (without weapons)” and “have been in a fight where you used a weapon (e.g., a knife).” In the 2018 survey, one item assessed participation in physical fighting: “have been in a fight.” At both time points, response options were *never* (0), *once* (1), *2–5 times* (2), *6–10 times* (3), and *11 times or more* (4). To generate comparable variables for the two time points, a single variable was computed for 2015, retaining the maximum score for the two items measuring physical fighting. For all regression analyses, physical fighting was dichotomized at both time points into no fights versus at least one fight. All other study measures were assessed identically in the two surveys.

#### Adult supervision

Five instruments assessed activities and situations with varying degree of adult supervision. First, parental supervision (Olweus 1989) was measured using three items on parents’ knowledge of their children’s social life: “My parents usually know where I am, and who I’m with, in my free time,” “My parents know most of the friends I hang out with in my free time,” and “My parents know my friends’ parents.” The response options were *not true at all* (1), *not very true* (2), *quite true* (3), and *very true* (4). Mean scores were computed, ranging from 1 to 4 (*α* = 0.74). Second, a mean score was generated by averaging six items measuring participation in the following organized leisure activities in the previous month: “sports club,” “youth club,” “religious organization,” “band, choir, orchestra,” “cultural school/music school,” and “other organization, team, association.” The response options were *never* (0), *1–2 times* (1), *3–4 times* (2), and *5 times or more* (3), returning a variable with a range from 0 to 3. Third, to assess the amount of time spent at home, the respondents’ indicated in a single item how many times in the previous week they had “been at home the whole evening,” with response options *never* (0), *once* (1), *2–5 times* (2), and *6 times or more* (3). Fourth, a single item with the same response options assessed how many times in the previous week the respondents had “spent the majority of the evening out with friends.” Finally, school truancy in the last 12 months was measured by a single question indicating the frequency of truancy, with response options *never* (0), *once* (1), *2–5 times* (2), *6–10 times* (3), and *11 times or more* (4).

#### Digital media use

A single item was used to assess how much time the respondents normally used outside of school on “activities in front of a screen (TV, computer, tablet, smartphone),” with response options *no time* (0), *less than 1* *h* (1), *1–2* *h* (2), *2–3* *h* (3), *3–4* *h* (4), *4–6* *h* (5), and *more than 6* *h* (6). A second single item assessed how much time the respondents spent daily on “social media (e.g., Facebook, Instagram, etc.),” with response options *no time* (0), *under 30* *min* (1), *30* *min to 1* *h* (2), *1–2* *h* (3), *2–3* *h* (4), and *more than 3* *h* (5).

#### Substance use

Alcohol intoxication and cannabis use were assessed by two single items from an instrument measuring the frequency of alcohol intoxication and illicit drug use in the previous 12 months, with response options *never* (0), *once* (1), *2–5 times* (2), *6–10 times* (3), and *11 times or more* (4).

#### Years of schooling

A single item measured the respondents’ years of schooling (range 8–13).

#### Socioeconomic background

The respondents’ socioeconomic background was measured by a composite score, averaging the score of three variables ranging from 0 to 3: (a) the number of parents having a university degree, (b) the number of books in the home of the respondents, and (c) the average score on the four-item Family Affluence Scale II (Currie et al. 2008). The instrument has been presented in detail in previous publications (Pedersen et al. 2018).

#### Migration background

Migration background was assessed using a single item separating those with two parents born outside of Norway from the remaining participants.

#### School grades

School grades in the subjects written Norwegian, English, and mathematics were assessed, and a mean score was computed (range 1–6).

#### Gender

The participants’ gender was assessed.

### Statistical Analyses

Changes in the prevalence of physical fighting from 2015 to 2018 were analyzed by means of cross tabulations and *χ*^2^ tests. Next, several analyses were conducted to investigate whether co-occurring changes in leisure time activities contributed to statistically account for the change in physical fighting. To be able to account for changes in physical fighting, the included variables had to fulfill three criteria: (1) changes had correspond to changes in physical fighting, (2) the variable had to correlate with physical fighting, and (3) the variable had to show a significant indirect effect in mediation analyses (Frøyland and von Soest 2018). To account for the possibility of gender-specific associations between physical fighting and the different leisure time activities, moderation analyses including a dummy variable for survey year, each of the leisure time activities, gender, and interaction terms between gender and the leisure time activities were conducted. The identification of significant interactions would imply a need for conducting gender separated analyses. Second, a series of linear regression analyses identified changes over time in the included leisure time activities. Third, all variables were correlated with physical fighting. Fourth, all leisure time activities that correlated with physical fighting and showed appropriate co-occurring changes were included one by one in separate probit regression analyses, together with a dummy variable for survey year. Whether the change in the leisure activity was significantly related to the change in physical fighting was assessed by means of mediation analyses under the counterfactual framework (VanderWeele 2015), which is the recommended framework for conducting mediation analyses with binary outcomes. Potential outcome probabilities were calculated based on the parameter estimates from the probit analyses, and results were presented as risk differences of these probabilities for the total effect (TE), the natural direct effect (NDE), and the natural indirect effect (NIE). The TE shows the increase in risk for physical fighting between the counterfactual outcomes of letting the total sample be from 2018 and allowing the mediator to change to the value from 2018 compared to letting the total sample be from 2015 and keeping the mediator value to the level from 2015. The NDE shows the increase in risk for physical fighting between the counterfactual outcomes of letting the total sample be from 2018, but keeping the mediator as it was in 2015, compared to letting the total sample be from 2015 and keeping the mediator as it was in 2015. The NIE shows the increase in risk for physical fighting between the counterfactual outcomes of letting the total sample be from 2018 and allowing the mediator to change to the value from 2018 compared to letting the total sample be from 2018 and keeping the mediator value to the level from 2015. Standard errors were estimated using the Delta method. The Delta method generally returns valid estimates in large samples (Muthén et al. 2016). Finally, all leisure time variables with a significant NIE in the bivariate analyses were included in multivariate analyses where the combined NIE of change in all the leisure time variables was calculated. The analyses were conducted based on recommended methods for analyzing the combined impact of multiple mediators on a binary outcome using structural equation models (Nguyen et al. 2016). To account for possible confounding, years of schooling, socioeconomic background, migration background, and school grades were included as control variables in all analyses. Mediation analyses were additionally conducted using the product-of-coefficients method (Hayes 2018), tables are included in the appendices.

The analyses were conducted using Mplus Version 8.3. Missing data were handled by the full information maximum likelihood procedure, thereby providing missing data routines that are considered to be state of the art (Schafer and Graham 2002). All analyses were also conducted using listwise deletion, yielding similar results. Due to the large sample size, only findings with p-values less than 0.01 were considered statistically significant.

## Results

The prevalence of physical fighting among Oslo adolescents increased significantly from 2015 to 2018 (see Table 1). In junior high school, the prevalence rates for boys increased from 31.4% in 2015 to 38.1% in 2018 and from 8.9% to 13.1% for girls. The rate of physical fighting was somewhat lower among students in senior high school at both time points. Among senior high school boys, 20.4% reported physical fighting in the previous 12 months in 2015, while 29.4% had participated in a fight in 2018. The prevalence rates for senior high school girls increased from 5.8% in 2015 to 8.5% in 2018. At both time points and in both junior and senior high school, about 50% of the boys who reported physical fighting had participated in one fight and 50% in more than one fight. Among girls, participation in only one fight in the previous 12 months was more common than participating in more than one fight. In general, the prevalence rates increased for all response options, indicating that both the proportion of adolescents participating in physical fighting and the frequency of such behavior increased. The prevalence rates were significantly higher for boys than for girls. Concerning the changes from 2015 to 2018 in the instrument measuring physical fighting, all but 7 of the respondents in 2015 that reported physical fighting with a weapon also reported fighting without a weapon, indicating that the two versions of the instrument to a large degree capture a corresponding group of respondents in the two surveys.

**Table 1:** Frequency of physical fighting in 2015 and 2018 in boys and girls in junior high school and senior high school

	Boys								Girls							
	Junior high school				Senior high school				Junior high school				Senior high school			
	2015		2018		2015		2018		2015		2018		2015		2018	
	*%*	*n*	*%*	*n*	*%*	*n*	*%*	*n*	*%*	*n*	*%*	*n*	*%*	*n*	*%*	*n*
No physical fighting	68.6	3,853	61.9	4,110	79.6	3,929	70.6	3,516	91.1	5,428	86.9	5,972	94.2	5,142	91.5	5,034
Once	15.9	894	18.0	1,197	10.4	515	14.2	709	5.4	321	7.7	529	3.9	211	5.4	299
2–5 times	10.9	611	13.7	910	7.4	364	10.1	504	2.7	162	3.6	250	1.4	79	2.1	116
6–10 times	2.1	118	2.4	162	1.4	68	1.9	97	0.4	25	0.8	53	0.2	12	0.4	20
11 times or more	2.6	144	3.9	262	1.2	61	3.1	156	0.4	23	1.0	72	0.2	12	0.5	30
Total	100.0	5,620	100.0	6,641	100.0	4,937	100.0	4,982	100.0	5,959	100.0	6,876	100.0	5,456	100.0	5,499

*Note.* All differences over time, gender, and school level were significant at *p* < 0.001

Initial moderation analyses revealed that the associations between physical fighting and eight out of the nine explanatory variables varied by gender (*p* < 0.01), thereby indicating a need for gender-specific analyses. The school levels were combined in the remaining analyses, with years of schooling, socioeconomic background, migration background, and school grades included as control variables. The surveys were conducted 3 years apart, and there is a substantial overlap of the samples as many of the junior high school participants in the first survey are attending senior high school in the second survey. Accordingly, all analyses were also conducted separately for junior and senior high school, returning similar results.

The proportion of girls did not differ between the two surveys (2015: 51.6%; 2018: 50.8%; *χ*^2^ = 2.835, *p* = 0.092). Table 2 shows additional descriptive statistics for all study variables for boys and girls in both surveys. The proportion of students with a migration background did not change neither for boys nor girls between the two surveys. The average years of schooling was slightly higher in 2015 than in 2018, while the average socioeconomic background and school grades increased slightly from 2015 to 2018.

**Table 2:** Descriptive statistics and change in study variables 2015–2018

	Boys				Girls				Total			
	*M (SD)*/%				*M (SD)*/%				*M (SD)*/%			
	2015 (*n* = 10,896)	2018 (*n* = 12,376)	Cohen’s *d*	*p*	2015 (*n* = 11,604)	2018 (*n* = 12,779)	Cohen’s *d*	*p*	2015 (*n* = 23,381)	2018 (*n* = 25,287)	Cohen’s *d*	*p*
Leisure time activities												
Parental monitoring (1–4)	3.11 (0.66)	3.13 (0.69)	0.03	0.138	3.23 (0.60)	3.27 (0.61)	0.07	0.364	3.18 (0.63)	3.20 (0.66)	0.04	0.442
Evenings at home (0–3)	1.79 (0.88)	1.84 (0.92)	0.06	<0.001	1.88 (0.80)	1.91 (0.83)	0.04	0.006	1.84 (0.84)	1.88 (0.80)	0.05	<0.001
Organized leisure activities (0–3)	0.55 (0.50)	0.59 (0.57)	0.07	<0.001	0.49 (0.49)	0.52 (0.53)	0.06	0.009	0.53 (0.53)	0.57 (0.60)	0.07	<0.001
Evenings out with friends (0–3)	1.01 (0.93)	1.07 (0.96)	0.06	<0.001	0.96 (0.90)	1.01 (0.90)	0.08	<0.001	0.99 (0.92)	1.04 (0.93)	0.05	<0.001
School truancy (0–4)	0.72 (1.17)	0.80 (1.21)	0.07	<0.001	0.74 (1.15)	0.73 (1.21)	0.01	<0.001	0.73 (1.16)	0.76 (1.17)	0.03	<0.001
Screen time (0–6)	3.52 (1.46)	3.86 (1.39)	0.24	<0.001	3.34 (1.42)	3.67 (1.34)	0.24	<0.001	3.42 (1.45)	3.76 (1.37)	0.24	<0.001
Social media use (0–5)	2.41 (1.49)	2.69 (1.49)	0.19	<0.001	3.10 (1.50)	3.45 (1.40)	0.24	<0.001	2.78 (1.53)	3.09 (1.49)	0.20	<0.001
Alcohol intoxication (0–4)	0.81 (1.37)	0.81 (1.37)	0.00	0.068	0.84 (1.35)	0.78 (1.31)	0.05	0.012	0.82 (1.36)	0.79 (1.34)	0.02	0.527
Cannabis use (0–4)	0.31 (0.91)	0.51 (1.17)	0.19	<0.001	0.17 (0.63)	0.23 (0.75)	0.09	<0.001	0.23 (0.78)	0.37 (0.98)	0.16	<0.001
Control variables												
Years of schooling (8–13)	10.33 (1.64)	10.23 (1.66)	0.06	<0.001	10.36 (1.68)	10.27 (1.67)	0.05	<0.001	10.33 (1.66)	10.36 (1.66)	0.05	<0.001
Socioeconomic background (0–3)	1.87 (0.76)	2.06 (0.66)	0.26	<0.001	1.93 (0.73)	2.07 (0.66)	0.21	<0.001	1.90 (0.74)	2.06 (0.66)	0.24	<0.001
Migration background (% yes)	32.7	33.6		0.162	34.3	34.1		0.713	33.9	33.9		0.974
School grades (1–6)	3.97 (0.89)	4.01 (0.90)	0.05	<0.001	4.09 (0.83)	4.19 (0.83)	0.11	<0.001	4.03 (0.86)	4.11 (0.87)	0.09	<0.001

*Note.* Difference tests for the leisure time activities were conducted by linear regression analyses with years of schooling, socioeconomic background, migration background, and school grades as control variables

The first step in identifying factors potentially relevant for understanding the increase in physical fighting was to analyze co-occurring changes in relevant leisure time activities; Table 2 shows the results. Factors related to an increased risk of physical fighting on an individual level in previous research should show co-occurring changes in the same direction as fighting, while factors related to a lessened risk should show opposing changes. Concerning adult supervision, the level of parental knowledge of adolescent activities and participation in organized activities increased among both boys and girls, making changes in these domains unsuitable for understanding the observed increase in physical fighting. On the other hand, the amount of time spent at home decreased for both genders, and a change in this manner of spending leisure time could be related to an increase in fighting. Turning to activities typically unsupervised by adults, both boys and girls reported spending more leisure time out with friends, highlighting this variable as potentially relevant for understanding the increase in physical fighting. Similarly, school truancy increased among boys, but it remained stable among girls. As expected, both screen time in general and the use of social media increased for both genders and were as such included in further analyses. Finally, the prevalence of alcohol intoxication remained unchanged among both boys and girls, but both genders reported a significant increase in their use of cannabis. A co-occurring change in cannabis use could therefore be relevant for understanding the increase in physical fighting. Summing up, co-occurring changes in evenings at home, evenings out with friends, screen time, social media use, and cannabis use remained potentially relevant for understanding the increase in physical fighting for both genders, while school truancy was relevant for boys only.

The second step in identifying factors potentially relevant for understanding the increase in fighting was to examine correlations between the included variables (see Table 3). As expected, factors that on an individual level are considered to decrease the risk of violence were negatively correlated with physical fighting, and factors considered to increase the risk correlated in the opposite direction. For boys, the highest correlations were observed for school truancy, cannabis use, and evenings out with friends (*r* = 0.20–0.28), whereas for girls, school truancy, cannabis use, and parental supervision correlated the highest with physical fighting (*r* = 0.13–0.23). Most correlations were in the range of small to medium effects according to the classification by Cohen (1988).

**Table 3:** Correlation matrix between all study variables: boys above the diagonal, girls below the diagonal

	1	2	3	4	5	6	7	8	9	10
1. Physical fighting (0–4)	–	−0.13**	−0.08**	0.12**	0.20**	0.28**	0.03**	0.14**	0.12**	0.21**
2. Parental monitoring (1–4)	−0.13**	–	0.00	0.13**	−0.05**	−0.23**	−0.11**	−0.09**	−0.17**	−0.20**
3. Evenings at home (0–3)	−0.02*	−0.01	–	−0.07**	−0.21**	−0.04**	0.21**	−0.07**	−0.09**	−0.07**
4. Organized leisure activities (0–3)	0.03**	0.11**	−0.07**	–	0.09**	−0.04**	−0.14**	0.04**	−0.10**	−0.06**
5. Evenings out with friends (0–3)	0.10**	−0.09**	−0.22**	0.01	–	0.19**	−0.04**	0.24**	0.28**	0.22**
6. School truancy (0–4)	0.23**	−0.29**	−0.04**	−0.12**	0.22**	–	0.14**	0.19**	0.37**	0.39**
7. Screen time (0–6)	0.10**	−0.13**	0.13**	−0.10**	0.09**	0.20**	–	0.26**	0.06**	0.08**
8. Social media use (0–5)	0.10**	−0.08**	−0.01	−0.07**	0.22**	0.20**	0.50**	–	0.19**	0.15**
9. Alcohol intoxication (0–4)	0.04**	−0.22**	−0.12**	−0.14**	0.29**	0.39**	0.08**	0.16**	–	0.58**
10. Cannabis use (0–4)	0.19**	−0.21**	−0.07**	−0.08**	0.17**	0.36**	0.08**	0.10**	0.46**	–

*p < 0.01; **p < 0.001

In a final step of analysis, all factors correlated with physical fighting and showing co-occurring changes in the appropriate direction were included in separate probit regression analyses together with survey year and the control variables measuring years of schooling, socioeconomic background, migration background, and school grades (see Table 4). Among boys, the analyses including evenings out with friends, school truancy, screen time, social media use, and cannabis use all returned significant NIE in the individual analyses, indicating that change in the respective variables were related to the change in physical fighting. Apart from school truancy, the same variables were related to the change in physical fighting among girls. In general, most of the included variables individually accounted for rather small parts of the observed increase in physical fighting, especially among girls.

**Table 4:** Results of probit regression analyses with physical fighting as dependent variable and year of survey and leisure time activities as predictors

	Total effect (TE)^a^		Natural direct effect (NDE)^b^		Natural indirect effect (NIE)^c^	
	*RD*	99% *CI*	*RD*	99% *CI*	*RD*	99% *CI*
Boys						
Evenings at home (0–3)	8.4%	[6.8, 10.0%]	8.6%	[7.1, 10.2%]	−0.2%	[−0.4, −0.1%]
Evenings out with friends (0–3)	8.4%	[6.8, 10.0%]	7.8%	[6.3, 9.4%]	0.6%	[0.2, 0.9%]
School truancy (0–4)	8.1%	[6.5, 9.7%]	6.7%	[5.2, 8.2%]	1.4%	[1.0, 1.8%]
Screen time (0–6)	8.4%	[6.8, 10.0%]	8.0%	[6.4, 9.6%]	0.4%	[0.1, 0.6%]
Social media use (0–5)	8.4%	[6.8, 10.0%]	7.1%	[5.6, 8.7%]	1.3%	[1.0, 1.5%]
Cannabis use (0–4)	8.2%	[6.6, 9.7%]	6.2%	[4.7, 7.8%]	2.0%	[1.6, 2.3%]
Girls						
Evenings at home (0–3)	3.8%	[2.9, 4.6%]	3.8%	[3.0, 4.7%]	0.0%	[–0.1, 0.0%]
Evenings out with friends (0–3)	3.7%	[2.9, 4.6%]	3.5%	[2.7, 4.4%]	0.2%	[0.1, 0.3%]
Screen time (0–6)	3.8%	[2.9, 4.6%]	3.2%	[2.4, 4.0%]	0.6%	[0.4, 0.7%]
Social media use (0–5)	3.8%	[3.0, 4.7%]	3.2%	[2.3, 4.0%]	0.7%	[0.5, 0.8%]
Cannabis use (0–4)	3.6%	[2.8, 4.4%]	3.2%	[2.4, 4.0%]	0.4%	[0.3, 0.5%]

*Note.* Years of schooling (centered), socioeconomic background (centered), migration background, and school grades (centered) were included as control variables in all analyses. Probabilities were calculated at the value 0 for all control variables. *RD*: risk difference; 99% *CI*: 99% confidence interval of *RD*

^a^TE shows the increase in risk for physical fighting between the counterfactual outcomes of letting the total sample be from 2015 and keeping the mediator value to the level from 2015 compared to letting the total sample be from 2015 and keeping the mediator value to the level from 2015

^b^NDE shows the increase in risk for physical fighting between the counterfactual outcomes of letting the total sample be from 2018, but keeping the mediator as it was in 2015, compared to letting the total sample be from 2015 and keeping the mediator as it was in 2015

^c^NIE shows the increase in risk for physical fighting between the counterfactual outcomes of letting the total sample be from 2018 and allowing the mediator to change to the value from 2018 compared to letting the total sample be from 2018 and keeping the mediator value to the level from 2015

In a final model, all variables showing significant NIE in the single variable analyses were included simultaneously in a regression analysis. For boys, the potential prevalence of physical fighting was estimated to be 24.6% (99% *CI*: 23.3–25.8%) had the whole sample been from 2015; 32.6% (99% *CI*: 31.2–34.0%) had the whole sample been from 2018 and the mediators were allowed to change; 29.6% (99% *CI*: 28.3–30.9%) had the whole sample been from 2018 but the mediators been kept as they were in 2015. Based on the potential outcomes, the following risk differences were calculated: TE_RD_ = 8.0% (99% *CI*: 6.5–9.6%); NDE_RD_ = 5.0% (99% *CI*: 3.5–6.5%); NIE_RD_ = 3.0% (99% *CI*: *2*.4–3.6%). The proportion of the change in physical fighting among boys mediated through change in the included leisure time activities was thus 37.5%.

For girls, the similar estimates of potential prevalence were 5.3% (99% *CI*: 4.7–5.9%) had the whole sample been from 2015; 9.0% (99% *CI*: 8.1–9.8%) had the whole sample been from 2018 and the mediators were allowed to change; 7.9% (99% *CI*: 7.1–8.6%) had the whole sample been from 2018 but the mediators been kept like they were in 2015. This equals the following risk differences: TE_RD_ = 3.6% (99% *CI*: 2.8–4.5%); NDE_RD_ = 2.5% (99% *CI*: 1.7–3.3%); NIE_RD_ = 1.1% (99% *CI*: 0.9–1.3%). In total, 30.6% of the increase in fighting among girls was mediated through changes in the included leisure time activities, but the initial risk for fighting was significantly lower than among boys.

Mediation analyses were also conducted using the product-of-coefficients method (Hayes 2018), yielding similar results. Results are presented in the appendices.

## Discussion

After several years of declines in violent behavior among adolescents (Arnett 2018), the present study identified an increase in physical fighting among Norwegian adolescents from 2015 to 2018 and investigated how this co-occurred with changes in leisure time habits. The most prominent changes for understanding the increase in physical fighting were co-occurring increases in time spent out with friends, digital media use, and cannabis use for both genders, as well as an increase in school truancy for boys. The amount of time spent at home decreased for both genders, but it was not related to the changes in physical fighting. Parental supervision and participation in organized leisure activities changed in the opposite direction of physical fighting, and the level of alcohol intoxication was stable in boys and declined in girls. As such, these factors were not of relevance for understanding the observed increase in physical fighting.

The increase in physical fighting among Oslo youth co-occurs with a not able increase in registered youth crime in the same area. According to police statistics (The City of Oslo and Oslo Police District 2019), the level of violent crime among adolescents under the age of 18 has not been higher on this side of the millennium than it was in 2018. The increase in violent crime in Oslo is more pronounced than what is found in other countries, but it is partly corroborated by recent increases in registered crime among young boys in Sweden (The Swedish National Council for Crime Prevention 2020) and violent crime in Denmark (Statistics Denmark 2020) and knife-related violence in the UK (Office for National Statistics 2020) in the general population. Even though crime statistics in several countries indicate a possible trend change in recent years, it is premature to conclude whether the observed increase in adolescent violence is part of a permanent trend of a higher level of adolescent misbehavior or just represents short-lasting fluctuations.

The present study hypothesized that three different domains related to adolescent leisure time are important for understanding changes in violent behavior: adult supervision, digital media use, and substance use. A notable finding is that the increase in physical fighting indeed co-occurred with an increase in time spent on activities normally happening outside of adult control, as measured by time spent out with friends and school truancy. This finding supports the notion that leisure time unsupervised by adults can be important for understanding changes in the rate of adolescent misbehavior on a societal level. Of note, study results are in line with theories such as the Routine Activity Approach, where a key component for understanding the occurrence of crime is a lack of capable guardians (Hollis et al. 2013). Similarly, others have suggested that increased socialization with delinquent peers may be a central aspect for understanding adolescent problem behavior (Hoeben et al. 2016). Previous research found that the amount of time that adolescents spend hanging out with friends has decreased significantly in the last two decades (Twenge 2017), which has been hypothesized as a contributing cause of the observed decline in problem behavior (Arnett 2018). Therefore, the fact that trend changes in spending leisure time out with friends co-occurs with increased rates of violent behavior is especially interesting. However, results also showed an increase in time spent participating in organized activities, which are typically under adult supervision. The increase in physical fighting might have been even larger if time spent in this manner had decreased as well.

Regarding digital media use, increases in overall screen time and in time spent on social media were related to the increase in physical fighting for both genders. This partly contradicts previous research suggesting that an increase in screen time contributes to a decline in problem behavior among adolescents (Arnett 2018). However, the pacifying effect of digital media is expected to disappear because digital media platforms become increasingly portable (Green 2016). Studies have also suggested that specific aspects of social media use, such as bullying and aggressive communication, can increase the risk of real-life violent behavior (Cannon et al. 2015). The co-occurring increases in physical fighting and both overall screen time and social media use may therefore be a result of the role of smartphones in modern media consumption, which enable adolescents to use digital media, and especially social media, outside of the home (Pew Research Center 2018). Following this, social media can be used in a context that facilitates physical fighting, such as in unsupervised communication with peer groups.

Finally, an increase in the use of cannabis remained one of the most prominent changes related to the increase in physical fighting. It is probable that an increased frequency of cannabis use may be related to an increasing number of adolescents socializing with delinquent peers (Haynie and Osgood 2005). In other words, the increase in cannabis use may be associated to the increase in fighting through processes where adolescents more often are socializing in environments that facilitate delinquent behaviors such as both cannabis use and physical fighting. This interpretation is in line with previous studies indicating that the association between cannabis use and violence is mainly a result of an antisocial lifestyle in general (Barthelemy et al. 2016).

The present study utilized two high-quality surveys covering about two out of three adolescents between the ages of 13 and 18 residing in the municipality of Oslo, Norway, thereby providing a solid base for examining co-occurring associations between adolescent leisure time activities and violent behavior. The close proximity in time of the surveys also facilitated an analysis of how fast-changing trends in digital media use were associated with adolescent physical fighting. Although co-occurring changes in the leisure time activities included in this study statistically could account for over 35% of the observed increase in physical fighting among boys and over 30% among girls, a sizeable portion of the increase in fighting was not accounted for by the included factors. A possible factor for understanding changes in the societal level of adolescent violence not explored in the present study is mental health problems, which have been found to be related to violent behavior (Dutton and Karakanta 2013) and have also increased among adolescents in recent years (Collishaw 2015). The acceptance of violent behavior may also vary over time, which can contribute to rising rates of violence among adolescents, but due to a lack of data, this remains mere speculation.

The study also has its limitations. First, due to the cross-sectional nature of the surveys, it was not possible to assess the temporal order of changes in physical fighting and the included leisure time factors. The possibility of reverse causation may be of particular importance for some of the included factors, such as cannabis use. Second, the study was based on two time points only, thereby restricting the analyses to linear change instead of more complex time trends. Third, as the analyses were based on self-reports only, there is some uncertainty concerning the accuracy of the responses, particularly when non-normative behavior such as physical fighting is assessed. Fourth, several of the included variables were single item measures and may as such have rather low reliability. Fifth, leisure time that adolescents spend at home and out with friends was assessed without providing information whether such activities were supervised by adults or not. As a result, considering such activities to be either supervised or unsupervised are based on implicit assumptions about what would be most common in these situations. Finally, even though physical fighting was assessed using identical initial wording in 2015 and 2018, the study is limited by the fact that physical fighting in 2015 was assessed using a combination of two items (physical fighting with and without a weapon), whereas only one overall item on physical fighting was used in 2018. The overall level of physical fighting in 2015 should not be influenced by using two items, as close to all respondents that reported fighting with weapons also reported fighting without. The respondents may still have interpreted physical fighting somewhat differently when asked explicitly about physical fighting with and without weapons, which might have influenced the reported level of fighting. Nevertheless, police reports from the same period corroborate the increase in physical fighting observed in the surveys, thereby strengthening the interpretations from the analyses. Apart from the instrument on physical fighting, all other items were measured identically in the two surveys.

Despite of these limitations, this study highlights the importance of considering leisure time activities in violence prevention work among adolescents. Understanding the role of leisure time activities for changes in adolescent violent behavior can aid designing programs for prevention of adolescent aggressive acts and thereby help to reduce such behavior. Of special importance could be providing access to adult-supervised leisure time activities for adolescents at risk of problem behavior. Among Oslo youth, the increase in fighting co-occurred with an increase in leisure time spent hanging out with friends, and access to leisure activities where adults are present might have mitigated the increase. Concerning digital media use, the use among adolescents will to all appearances continue to increase, making it paramount for both youth workers and other adults to develop strategies to help children and adolescents navigate this new territory in a sensible way.

## Conclusion

The present study used two population-based cross-sectional surveys among Norwegian adolescents to provide novel information about recent changes in the societal level of physical fighting and how such changes are related to co-occurring changes in leisure time activities. The study finds a significant increase in physical fighting among Norwegian adolescents from 2015 to 2018, among both boys and girls and in junior and senior high school. Several factors related to adolescent leisure time contributed to understanding the change in physical fighting: co-occurring increases in time spent unsupervised by adults, digital media use, and use of cannabis. The study thereby provides important information about how adolescents’ leisure activities are interwoven with adolescent problem behavior such as physical fighting. Of particular interest is the observed association between increasing digital media use and rising levels of physical fighting. This finding indicates that the recent years’ proliferation of smartphone use, by enabling adolescents to use digital media outside of the home and without adult presence, may have altered the previously observed association between digital media use and low levels of problems behavior.

## Appendix 1

Logistic regression analyses with physical fighting as dependent variable and year of survey and leisure time variables as predictors (boys). Mediation analyses are based on the product-of-coefficients method.

**Table:**

	Relationship between predictor variable and physical fighting				Change in physical fighting from 2015 to 2018^a^	
	*OR*	99% *CI*	Indirect effect^b^	99% *CI*	*OR*	99% *CI*
Baseline model (without predictors)					1.51**	[1.40, 1.63]
Model 1 (separate analyses for each predictor)						
Evenings at home (0–3)	0.82**	[0.78, 0.85]	−0.01**	[–0.02, 0.00]	1.54**	[1.42, 1.66]
Evenings out with friends (0–3)	1.56**	[1.49, 1.62]	0.03**	[0.01, 0.04]	1.49**	[1.38, 1.62]
School truancy (0–4)	1.62**	[1.56, 1.68]	0.07**	[0.05, 0.09]	1.43**	[1.32, 1.54]
Screen time (0–6)	1.05**	[1.02, 1.08]	0.02**	[0.01, 0.03]	1.49**	[1.37, 1.60]
Social media use (0–5)	1.22**	[1.18, 1.25]	0.06**	[0.05, 0.07]	1.44**	[1.32, 1.55]
Cannabis use (0–4)	1.50**	[1.44, 1.57]	0.09**	[0.08, 0.11]	1.38**	[1.28, 1.49]
Model 2 (all variables showing co-occurring changes with physical fighting included simultaneously)^c^						
Evenings out with friends (0–3)	1.38**	[1.31, 1.44]	0.02**	[0.01, 0.03]		
School truancy (0–4)	1.48**	[1.43, 1.59]	0.03**	[0.01, 0.05]		
Screen time (0–6)	0.99	[0.96, 1.02]	0.00	[–0.01, 0.01]		
Social media use (0–5)	1.11**	[1.08, 1.15]	0.03**	[0.02, 0.04]		
Cannabis use (0–4)	1.25**	[1.19, 1.31]	0.05**	[0.03, 0.06]	1.32**	[1.21, 1.43]

*Note.* Confidence intervals were calculated based on 1,000 bootstrap samples. Years of schooling, socioeconomic background, migration background, and school grades were included as control variables in all analyses. *OR*: odds ratio; 99% *CI*: 99% confidence interval of *OR*

**p* < 0.01; ***p* < 0.001

^a^The change in physical fighting from 2015 to 2018 was estimated by the *OR* of the association between survey year and physical fighting

^b^Indirect effect (mediation effect) of the association between survey year and physical fighting via leisure time variables. Indirect effects provide information about whether change in the included leisure time variables statistically reduce the estimate of change in physical fighting from 2015 to 2018

^c^The total indirect effect in Model 2 was estimated to 0.13 (*p* < 0.001, 99% *CI* [0.10, 0.17]), or 32.7% of the total effect

## Appendix 2

Logistic regression analyses with physical fighting as dependent variable and year of survey and leisure time variables as predictors (girls). Mediation analyses are based on the product-of-coefficients method.

**Table:**

	Relationship between predictor variable and physical fighting				Change in physical fighting from 2015 to 2018^a^	
	*OR*	99% *CI*^c^	Indirect effect^b^	99% *CI*	*OR*	99% *CI*
Baseline model (without predictors)					1.74**	[1.52, 1.97]
Model 1 (separate analyses for each predictor)						
Evenings at home (0–3)	0.89**	[0.83, 0.95]	0.00	[−0.01, 0.00]	1.75**	[1.53, 1.98]
Evenings out with friends (0–3)	1.50**	[1.40, 1.61]	0.02**	[0.01, 0.04]	1.70**	[1.48, 1.91]
Screen time (0–6)	1.22**	[1.16, 1.29]	0.07**	[0.06, 0.10]	1.62**	[1.41, 1.83]
Social media use (0–5)	1.25**	[1.18, 1.32]	0.09**	[0.06, 0.11]	1.63**	[1.41, 1.84]
Cannabis use (0–4)	1.77**	[1.65, 1.90]	0.05**	[0.03, 0.06]	1.63**	[1.41, 1.84]
Model 2 (all variables showing co-occurring changes with physical fighting included simultaneously)^c^						
Evenings out with friends (0–3)	1.36**	[1.27, 1.45]	0.02**	[0.01, 0.03]		
Screen time (0–6)	1.12**	[1.05, 1.19]	0.04**	[0.02, 0.06]		
Social media use (0–5)	1.11**	[1.04, 1.17]	0.04**	[0.01, 0.06]		
Cannabis use (0–4)	1.65**	[1.52, 1.77]	0.03**	[0.02, 0.05]	1.49**	[1.30, 1.68]

*Note.* Confidence intervals were calculated based on 1,000 bootstrap samples. Years of schooling, socioeconomic background, migration background, and school grades were included as control variables in all analyses. *OR*: odds ratio; 99% *CI*: 99% confidence interval of *OR*

**p* < 0.01, ***p* < 0.001

^a^The change in physical fighting from 2015 to 2018 was estimated by the *OR* of the association between survey year and physical fighting

^b^Indirect effect (mediation effect) of the association between survey year and physical fighting via leisure time variables. Indirect effects provide information about whether change in the included leisure time variables statistically reduce the estimate of change in physical fighting from 2015 to 2018

^c^The total indirect effect in Model 2 was estimated to 0.06 (*p* < 0.001, *99% CI* [0.02, 0.10]), or 12.6% of the total effect
